# BK-Virus-Induced Hemorrhagic Cystitis in a Patient With Graft-Versus-Host Disease

**DOI:** 10.7759/cureus.35413

**Published:** 2023-02-24

**Authors:** Mohamed Zakee Mohamed Jiffry, Napat Rangsipat, Daniel Tabares, Aimal Khan, Teena Thomas

**Affiliations:** 1 Internal Medicine, Danbury Hospital, Danbury, USA; 2 Medicine, American University of the Caribbean, Cupecoy, SXM

**Keywords:** malignant hematology, allogeneic bone marrow transplant, graft vs host disease, hemorrhagic cystitis, bk polyomavirus

## Abstract

The BK virus is a polyomavirus known to particularly affect transplant recipients. An important complication caused by BK virus infection in bone marrow transplant patients is hemorrhagic cystitis. We present a 31-year-old male with a history of bone marrow transplantation complicated by graft-versus-host disease (GVHD) and was diagnosed with BK virus-related hemorrhagic cystitis. He presented with gross hematuria and suprapubic and penile pain for one week. He has a significant past medical history of acute B-cell lymphocytic leukemia for which he has successfully undergone allogenic bone marrow transplantation, which was complicated by GVHD. Imaging revealed significant bladder wall thickening which prompted an evaluation for BK virus-induced hemorrhagic cystitis. A urinary specimen was sent for BK virus polymerase chain reaction (PCR) which was strongly positive, confirming the infection. He was managed supportively throughout his hospitalization and improved with symptomatic management alone. Our case demonstrates one of the main complications caused by the BK virus in allogeneic bone marrow transplant patients in the setting of GVHD and is an important differential to keep in mind when treating patients presenting with hematuria after bone marrow transplantation.

## Introduction

The BK virus, so named after the initials of the patient the virus was isolated from, is a polyomavirus known to particularly affect post-transplant patients [1]. Transmission of the organism occurs person-to-person, with accumulating evidence suggesting oral and/or respiratory transmission being strongly implicated [2]. Following initial infection which is usually asymptomatic, viraemic spread occurs to other tissues and organs, principally the urinary tract in the case of the BK virus. It is estimated that up to 90% of adults are seropositive for the BK virus which remains latent in the urinary tract and can be reactivated when the host is immunocompromised [3]. Although the BK virus is the primary cause of polyomavirus-associated nephropathy in renal transplant recipients, another important complication caused by BK virus infection in patients with hematopoietic cell transplantation following engraftment is hemorrhagic cystitis. The BK virus-related hemorrhagic cystitis is estimated to complicate 5% to 25% of allogeneic hematopoietic cell transplants [4]. We present a 31-year-old male with a history of bone marrow transplantation complicated by graft-versus-host-disease (GVHD) and diagnosed with BK virus-related hemorrhagic cystitis.

## Case presentation

A 31-year-old male with a past medical history of allogeneic bone marrow transplantation for B-cell acute lymphoblastic leukemia 10 months ago complicated by GVHD presented to the emergency department complaining of worsening suprapubic and penile pain associated with bloody urine. He stated that his symptoms had been ongoing for eight days prior to the current presentation, and noted blood throughout the stream every time he urinates. He had been taking oxycodone 20 mg every two hours with minimal improvement in his pain. He did report one episode of fever on the day prior to the presentation. He denied feeling nauseous or having episodes of vomiting. He denied flank pain or tenderness. Examination revealed an uncomfortable-appearing male with normal vital signs. Cardiovascular and respiratory exams were unremarkable. An abdominal exam revealed some suprapubic tenderness and bloody urine was noted in his urine bag.

Two years ago, the patient was diagnosed with Philadelphia chromosome-positive acute B-cell lymphocytic leukemia. He completed a course of eight cycles of hyper-cyclophosphamide, vincristine sulfate, adriamycin, and dexamethasone (CVAD) with successful remission induction. He was subsequently referred for haploidentical allogeneic bone marrow transplantation which he successfully underwent. An HLA-matched donor was unavailable as he was an undocumented immigrant. Post-transplant, the patient was maintained on sirolimus and imatinib as well as antimicrobial prophylaxis with acyclovir, fluconazole, and letermovir. His post-transplant course was complicated by the development of chronic GVHD five months after his transplant after he presented with mucosal lesions, odynophagia, and generalized swelling. His GVHD was deemed low grade and it was decided against treatment with immunosuppressive therapy.

A urinalysis was obtained which showed turbid-appearing orange urine with 3+ blood and 3+ protein. Greater than 50 RBCs and 3-9 WBCs were also noted. No significant bacteriuria, crystals, or casts were appreciated. Basic serum chemistry and a complete blood count were within normal limits. A urine culture obtained for suspicion of infection did not reveal any significant growth.

**Figure 1:**
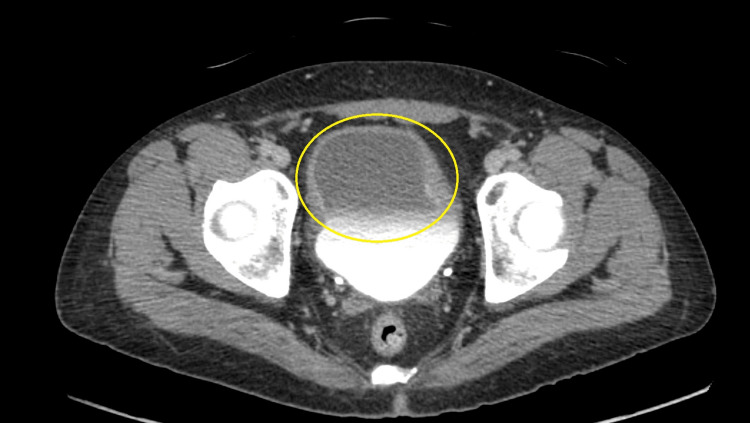
CT urogram of the patient The yellow circle marks the extensive and asymmetric bladder wall thickening noted

A CT urogram with contrast study revealed extensive thickening of the bladder wall (Figure 1). No enhancing masses were noted in the kidneys and no renal or ureteric calculi were appreciated. The prostate and the seminal vesicles were normal in appearance.

Although the patient was empirically managed with IV ceftriaxone for suspicion of infection, his negative cultures prompted concern for possible BK virus infection. A urology consultation was obtained for cystoscopy and biopsy. Active bleeding from the right lateral wall of his bladder was noted during the procedure, and electrocautery was applied to these areas with good hemostasis being achieved. Microscopic and macroscopic descriptions from biopsy sections showed reactive fibroblastic tissue and acute and chronic inflammation with no overt evidence of viral inclusions with cytomegalovirus (CMV) stain. The viral culture was also negative from the biopsy specimen.

A quantitative polymerase chain reaction (PCR) for the BK virus from his blood was sent and although BK viremia was detected, it was unable to be quantified owing to a low viral load below 390 copies per milliliter. Subsequently, a urinary specimen was sent for BK virus detection by PCR, which confirmed BK virus infection with 8,170,000 copies per mL being detected.

In consultation with his transplant physicians, it was decided against specific therapy for his infection with cidofovir and to continue supportive care alone. For his pain, he was continued on oxycodone and high-strength Tylenol. He was given Ditropan and Pyridium to assist with symptomatic relief as well. Post-procedure, he continued to improve with respect to the hematuria and pain and was subsequently discharged the following day.

## Discussion

The BK virus belongs to the polyomavirus family that is commonly acquired in childhood with a prevalence rate of up to 80% to 90% in adults [5]. The virus becomes latent in the urinary tract after infection and can be activated once the host becomes immunocompromised such as after transplantation. One of the main complications of the BK virus is hemorrhagic cystitis, which is seen in 5% to 15% of allogeneic hematopoietic stem cell transplants.

Our patient was diagnosed with B-cell acute lymphoblastic leukemia (ALL) for which he underwent a haploidentical bone marrow transplant. Annually, there are about 5000 patients diagnosed with ALL, and 1400 deaths are attributed to the disease. Despite their high remission rates of 80% to 90% for ALL, the overall survival rate is low in the adult population (30% to 40%) [1]. Survival rates in adult ALL have improved with better management of the complications of those undergoing allogeneic hematopoietic cell transplantation (alloHCT) such as GVHD and infection. The incidence of Philadelphia chromosome positivity (Ph+) in acute lymphoblastic leukemia (ALL) ranges from 15% to 30%, with a higher incidence in older patients (up to 50% in elderly patients) [6]. The prognosis for Ph+ ALL is poor and allogeneic hematopoietic stem cell transplantation (alloHCT) is the only potential curative option. Our patient was 31 years old and otherwise healthy, and as such he was considered a very good candidate for allogeneic hematopoietic stem cell transplantation.

In a retrospective study looking at patients who received hematopoietic stem cell transplants between 2016 and 2020, risk factors for BK virus-induced hemorrhagic cystitis, acute graft-versus-host disease (aGVHD), and others were discussed [7]. The study concludes that stem cell source (peripheral blood or peripheral blood combined with bone marrow), donor age, human leukocyte antigen (HLA) match, busulfan administration, anti-thymocyte globulin (ATG), total body irradiation, CMV in urine, BK virus in urine, and aGVHD were statistically significant risk factors in the regression analysis for the development of BK virus-associated hemorrhagic cystitis. Anti-thymocyte globulin, urine CMV, and urine BK virus were independent significant risk factors in this multivariate analysis. The receiver operating characteristic (ROC) curve analysis showed that when the BK virus load was >1x10^7 copies/mL, the risk of hemorrhagic cystitis was significantly increased. Another retrospective study linked BK virus hemorrhagic cystitis with GVHD and a longer hospitalization [8]. 

The incidence of aGVHD in patients with hematopoietic cell transplantation from HLA-matched siblings is 50% but the incidence is higher in unmatched donors [9]. Our patient had a haploidentical bone marrow transplant which put him at higher risk for GVHD and infections. While the incidence of chronic GVHD ranges from 6% to 80%, the morbidity and mortality due to GVHD after having hematopoietic cell transplantation is more than 10% [9].

The European Conference on Infections in Leukemia (ECIL) provides a guideline for BK virus-related hemorrhagic cystitis. The diagnostic criteria include clinical features of cystitis, grade 2 or higher hematuria, and BK viral viruria ≥ 7log10/mL. Hematuria grades 2, 3 and 4 include macroscopic hematuria, macroscopic hematuria with clots, and macroscopic hematuria with clots and renal dysfunction due to urinary obstruction, respectively [10].

The BK virus can be detected in both blood and urine. The BK viral viruria mostly precedes viremia and also resolves after the resolution of the viremia. Viral load in the urine is usually higher than in the blood making it a more sensitive protocol for detection [10]. The positive predictive value (PPV) of viremia, however, is between 50% to 60% which is higher than that of viruria at 40% [11]. In rare cases, BK viremia may be found without urine according to at least one study [10]. The screening recommendations for the BK virus vary by institution, and some institutions may prefer to do serum screening due to higher PPV while others may screen based on urine PCR and only do plasma screening if urine viral load rises above the threshold. Our case shows significantly higher copies of the BK virus in urinary specimens compared to blood samples. 

There is currently no standard and approved treatment protocol for BK virus-related hemorrhagic cystitis [12]. Most cases of BK virus-related hemorrhagic cystitis are mild and can be treated with supportive care as was done in our case, but severe cases may require further measures. Patients treated with immunosuppressant medications for other indications such as GVHD may warrant dose reduction or outright discontinuation in the setting of severe BK virus-related hemorrhagic cystitis. There are no specific antiviral drugs with strong evidence of efficacy against the BK virus. Cidofovir, brincidofovir, leflunomide, and quinolone antibiotics have demonstrated activity against viral replication and have been used therapeutically. A study evaluating the efficacy of intravesicular cidofovir for treating BK virus-related hemorrhagic cystitis demonstrated symptomatic improvement in 88% of patients with BK virus hemorrhagic cystitis, although bladder spasms were reported in 12% of the patients [12,13]. Further studies are needed to determine optimal treatment strategies for BK virus-related hemorrhagic cystitis.

## Conclusions

This case demonstrates one of the main complications caused by the BK virus in allogeneic bone marrow transplant patients in the setting of GVHD. It is important to keep this in mind when treating patients with GVHD after a bone marrow transplant since they are at increased risk for hemorrhagic cystitis. 

The overall management for patients with BK virus hemorrhagic cystitis depends on disease severity with mild cases only requiring conservative treatment. Although some studies report the use of cidofovir in the management of BK virus hemorrhagic cystitis, further studies are needed to determine the optimal treatment strategy for BK virus reactivation.
